# Splenic Artery Hemorrhage as a Rare Delayed Complication of Percutaneous Endoscopic Gastrostomy

**DOI:** 10.14309/crj.0000000000002206

**Published:** 2026-06-30

**Authors:** Joshua E. Pagán-Busigó, Ethan Shamsian, Rohan Karkra, Ayyad Mohammed, Kranthi Mandava, Salil Chowdhury, Gokturk Suut

**Affiliations:** 1 Department of Internal Medicine, Rutgers New Jersey Medical School, Newark, NJ; 2 Division of Gastroenterology and Hepatology, Rutgers New Jersey Medical School, Newark, NJ

**Keywords:** percutaneous endoscopic gastrostomy, gastrointestinal hemorrhage, splenic artery injury, endovascular embolization

## Abstract

Percutaneous endoscopic gastrostomy (PEG) is commonly used for long-term enteral access in patients with dysphagia. We report a 67-year-old man with laryngeal carcinoma, prior stroke, and severe dysphagia who developed delayed hemorrhage from a branch of the splenic artery 7 days after PEG placement. He presented with abdominal pain, bleeding through the PEG tube, profound anemia, and hemodynamic instability. Imaging showed hemorrhage in the lesser sac, and angiography confirmed active splenic artery branch bleeding. Endovascular embolization achieved hemostasis. This rare complication is notable for its delayed presentation and favorable outcome after prompt intervention.

## INTRODUCTION

Percutaneous endoscopic gastrostomy (PEG) is one of the most frequently performed procedures for establishing long-term enteral access in patients with impaired swallowing and poor oral intake. In the United States alone, approximately 150,000 to 200,000 PEGs are placed annually, and the worldwide numbers are substantially higher.^1^ Indications for PEG placement primarily include disorders that impair the swallowing mechanism. The most common indications are neurologic dysphagia (such as stroke, amyotrophic lateral sclerosis, and traumatic brain injury), head and neck malignancies, and conditions in which patients are unable to meet their caloric requirements due to chronic medical illnesses or prolonged critical care needs. PEG is also indicated in cases of anatomical, inflammatory, or functional obstruction of the upper gastrointestinal tract, where adequate oral intake is not feasible.^2,3^

Although PEG is generally considered safe, it is not without risk. Overall complication rates range from 8% to 30%, with major complications reported in fewer than 3% of cases.^4^ Common minor complications include peristomal infection, tube leakage, and dislodgement. Major adverse events, although rare, include gastrointestinal perforation, buried bumper syndrome and vascular or visceral injury.^5^ Intra-abdominal hemorrhage has been reported in fewer than 1% of cases, and bleeding of arterial origin is exceedingly rare but potentially fatal.^6^ This report presents a case of delayed splenic artery hemorrhage after PEG placement, underscoring the need for clinical vigilance and rapid multidisciplinary management in the setting of unexplained abdominal pain, anemia, or bleeding after PEG.

## CASE REPORT

A 67-year-old man with a history of laryngeal carcinoma status postradiotherapy, emphysema, asthma, and prior ischemic stroke presented with a 4-day history of weakness, productive cough, and fever and was found to have sepsis secondary to aspiration pneumonia with bacteremia. After treatment with intravenous antibiotics and fluids, a modified barium swallow demonstrated severe oropharyngeal dysphagia, and a PEG was placed for long-term enteral nutrition. Before PEG placement, hemoglobin was 9.7 g/dL, compared with a baseline of 12.0 g/dL. Platelet count ranged from 250 to 350 ×10^3^/µL and international normalized ratio was 1.1, consistent with the patient's baseline. He was receiving aspirin 81 mg daily and prophylactic enoxaparin, with no history of chronic liver disease or therapeutic anticoagulation. A computed tomography scan obtained on admission before PEG placement showed nonspecific esophageal mural thickening, with no bowel obstruction, free air, or reported vascular abnormality. However, no dedicated preprocedural vascular imaging had been performed.

The morning after PEG placement, he had one self-limited episode of small-volume blood-streaked emesis without recurrence. Over the following week, hemoglobin decreased and fluctuated between 7.5 and 8.5 g/dL before acutely dropping to 5.4 g/dL 7 days after PEG. At that time, he developed diffuse abdominal pain, blood clots in the PEG tube, and profound hypotension. Examination was notable for blood within the PEG tube and tenderness around the insertion site. The abdomen was nondistended with preserved bowel sounds, and mental status remained at baseline. At the time of deterioration, coagulation studies remained at baseline and platelet count was preserved without thrombocytopenia. He required 3 units of packed red blood cells, 1 unit of fresh frozen plasma, intravenous fluids, vasopressor support, and intensive care unit admission. Computed tomography angiography demonstrated intra-abdominal hemorrhage in the lesser sac (Figure 1), and mesenteric angiography showed active bleeding from a branch of the splenic artery (Figure 2), which was successfully embolized. He subsequently stabilized, tolerated enteral feeding, and was discharged after a 16-day hospitalization.

**Figure 1.**
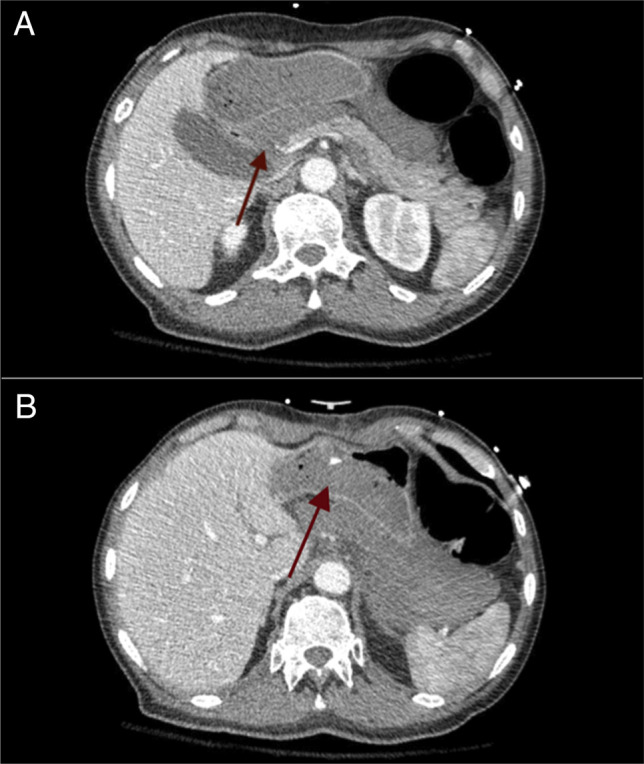
(A) Computed tomography angiography of the abdomen revealing a hemorrhagic fluid collection (red arrow) within the lesser sac, interposed between the stomach and the pancreas. (B) 0.7-cm hyperattenuation (red arrow) along the posterior margin of the gastrostomy tube seen on the venous phase, which may represent active contrast extravasation.

**Figure 2.**
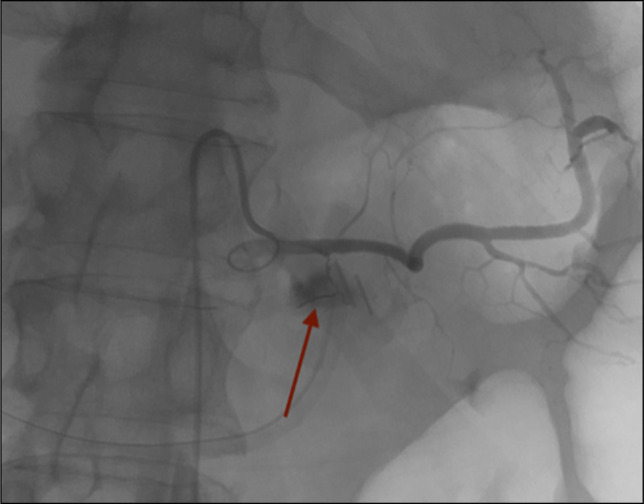
Mesenteric angiography of the abdomen demonstrating a focal blush of contrast (red arrow), consistent with active extravasation from a small branch of the splenic artery.

## DISCUSSION

This case highlights a rare presentation of delayed hemorrhagic shock after PEG placement, caused by splenic artery branch injury. Although PEG is widely performed and generally safe, vascular complications can be life-threatening and may present days after the procedure. In this patient, CT angiography was essential in the rapid identification of intra-abdominal bleeding, while endovascular intervention successfully repaired the iatrogenic injury. According to the adverse events in gastrointestinal endoscopy classification, this adverse event is most consistent with grade IVa severity because the patient developed hemorrhagic shock requiring vasopressor support and intensive care unit-level care, in addition to radiologic intervention for definitive hemostasis.^7^

To the best of our knowledge, this is the first documented case of delayed hemorrhagic shock from rupture of a splenic artery branch following PEG placement that was successfully managed with angiographic embolization. Previously reported PEG-associated vascular complications have occurred predominantly in older adults and have most often presented as hemoperitoneum or severe hemorrhage with shock, illustrating the potential for clinically significant injury involving both arterial and venous structures.^8–16^ Reported vascular injuries have involved the gastroepiploic, gastric, superior mesenteric, splenic, and abdominal wall vessels. Across published cases, the timing of hemorrhage has ranged from within the first hour to several days after PEG placement, and management has varied from endoscopic therapy and exploratory laparotomy to transcatheter arterial embolization, with outcomes ranging from successful hemostasis to death. Notably, previously reported splenic artery involvement was associated with early fatal hemorrhage, whereas our patient developed a later presentation from a splenic artery branch injury and survived after prompt recognition, imaging localization, and transcatheter embolization. A summary of previously reported PEG-associated vascular complications and the present case is presented in Table 1.

**Table 1.** Reported hemorrhagic vascular complications after PEG placement

Author, year	Age, sex	Bleeding complication	Involved vessel	Time to bleeding after PEG	Management	Outcome
Lewis et al, 1999	68, Female	Hemoperitoneum with shock	Right and left gastroepiploic arteries	5 d	TAE	Hemostasis achieved
Lau and Lai, 2001	93, Female	Retroperitoneal hemorrhage with shock	Splenic and superior mesenteric veins	2.5 h	EL	Fatal
Bordes et al, 2008	59, Male	Hemoperitoneum with shock	Unspecified gastric artery	1 h	EL with suturing	Hemostasis achieved
Smale et al, 2009	83, Male	Hemoperitoneum with shock	Splenic artery	7 h	EL	Fatal
Bunai et al, 2009	77, Female	Hemoperitoneum with shock	Left gastric artery	50 min	TAE	Fatal
Lee et al, 2018	64, Male	Hemoperitoneum with traumatic pancreatitis	Superior mesenteric artery	1 d	TAE	Hemostasis achieved
Enofe et al, 2019	94, Male	Gastrointestinal bleeding without shock	Presumed anterior abdominal wall vessel or gastric vessel	2 d	EGD with endoscopic clipping	Hemostasis achieved
Brazee et al, 2023	78, Male	Hemoperitoneum with shock	Right gastric artery	4 d	TAE	Hemostasis achieved
Ohri et al, 2025	63, Male	Hemoperitoneum with shock	Right gastroepiploic artery	12 h	TAE and EL with gastrectomy and jejunostomy	Hemostasis achieved
Present case	67, Male	Hemoperitoneum with shock	Splenic artery branch	7 d	TAE	Hemostasis achieved

EGD, esophagogastroduodenoscopy; EL, exploratory laparotomy; PEG, percutaneous endoscopic gastrostomy; TAE, transcatheter arterial embolization.

Clinical presentation may be nonspecific and include hypotension, tachycardia, abdominal pain, intraluminal bleeding, or an abrupt hemoglobin decline. In our case, isolated blood-streaked emesis on postprocedural day 1 was initially felt to represent minor mucosal irritation given its small volume and lack of recurrence, although an early sentinel bleed cannot be completely excluded. This case demonstrates that delayed hemorrhage can occur even 1 week after PEG placement and should remain in the differential when unexplained anemia, abdominal pain, or hemodynamic instability develops. Prompt imaging, resuscitation, and endovascular intervention may be lifesaving in these rare but life-threatening complications.^17–19^

## DISCLOSURES

Author contributions: JE Pagán-Busigó: Substantial contributions to the conception or design of the work; the acquisition, analysis, and interpretation of data for the work; and original drafting of the work. E. Shamsian: Drafting parts of the work; revising the work critically for important intellectual content. R. Karkra: Drafting parts of the work; revising the work critically for important intellectual content. A. Mohammed: Drafting parts of the work; revising the work critically for important intellectual content. K. Mandava: Drafting parts of the work. S.Chowdhury: Revising the work critically for important intellectual content. G. Suut: Substantial contributions to the conception or design of the work; the analysis, and interpretation of data for the work; and revising it critically for important intellectual content. G. Suut is the article guarantor.

Financial disclosure: None to report.

Previous presentation: This case was presented at the American College of Gastroenterology Annual Scientific Meeting, October 28, 2025; Phoenix, Arizona.

Informed consent was obtained for this case report.

## ABBREVIATIONS:

AGREE: Adverse Events in Gastrointestinal Endoscopy
EGD: esophagogastroduodenoscopy
EL: exploratory laparotomy
PEG: percutaneous endoscopic gastrostomy
TAE: transcatheter arterial embolization
